# An online survival predictor in glioma patients using machine learning based on WHO CNS5 data

**DOI:** 10.3389/fneur.2023.1179761

**Published:** 2023-05-19

**Authors:** Liguo Ye, Lingui Gu, Zhiyao Zheng, Xin Zhang, Hao Xing, Xiaopeng Guo, Wenlin Chen, Yaning Wang, Yuekun Wang, Tingyu Liang, Hai Wang, Yilin Li, Shanmu Jin, Yixin Shi, Delin Liu, Tianrui Yang, Qianshu Liu, Congcong Deng, Yu Wang, Wenbin Ma

**Affiliations:** ^1^Department of Neurosurgery, Center for Malignant Brain Tumors, National Glioma MDT Alliance, Peking Union Medical College Hospital, Chinese Academy of Medical Sciences and Peking Union Medical College, Beijing, China; ^2^Department of Neurosurgery, Beijing Tiantan Hospital, Capital Medical University, Beijing, China; ^3^Research Unit of Accurate Diagnosis, Treatment, and Translational Medicine of Brain Tumors (No. 2019RU011), Chinese Academy of Medical Sciences, Beijing, China; ^4^China Anti-Cancer Association Specialty Committee of Glioma, Beijing, China; ^5^4+4 Medical Doctor Program, Chinese Academy of Medical Sciences and Peking Union Medical College, Beijing, China; ^6^Eight-year Medical Doctor Program, Chinese Academy of Medical Sciences and Peking Union Medical College, Beijing, China

**Keywords:** glioma, WHO CNS5, machine learning, predictive analytics, prognosis

## Abstract

**Background:**

The World Health Organization (WHO) CNS5 classification system highlights the significance of molecular biomarkers in providing meaningful prognostic and therapeutic information for gliomas. However, predicting individual patient survival remains challenging due to the lack of integrated quantitative assessment tools. In this study, we aimed to design a WHO CNS5-related risk signature to predict the overall survival (OS) rate of glioma patients using machine learning algorithms.

**Methods:**

We extracted data from patients who underwent an operation for histopathologically confirmed glioma from our hospital database (2011–2022) and split them into a training and hold-out test set in a 7/3 ratio. We used biological markers related to WHO CNS5, clinical data (age, sex, and WHO grade), and prognosis follow-up information to identify prognostic factors and construct a predictive dynamic nomograph to predict the survival rate of glioma patients using 4 kinds machine learning algorithms (RF, SVM, XGB, and GLM).

**Results:**

A total of 198 patients with complete WHO5 molecular data and follow-up information were included in the study. The median OS time of all patients was 29.77 [95% confidence interval (CI): 21.19–38.34] months. Age, FGFR2, IDH1, CDK4, CDK6, KIT, and CDKN2A were considered vital indicators related to the prognosis and OS time of glioma. To better predict the prognosis of glioma patients, we constructed a WHO5-related risk signature and nomogram. The AUC values of the ROC curves of the nomogram for predicting the 1, 3, and 5-year OS were 0.849, 0.835, and 0.821 in training set, and, 0.844, 0.943, and 0.959 in validation set. The calibration plot confirmed the reliability of the nomogram, and the c-index was 0.742 in training set and 0.775 in validation set. Additionally, our nomogram showed a superior net benefit across a broader scale of threshold probabilities in decision curve analysis. Therefore, we selected it as the backend for the online survival prediction tool (Glioma Survival Calculator, https://who5pumch.shinyapps.io/DynNomapp/), which can calculate the survival probability for a specific time of the patients.

**Conclusion:**

An online prognosis predictor based on WHO5-related biomarkers was constructed. This therapeutically promising tool may increase the precision of forecast therapy outcomes and assess prognosis.

## Introduction

Gliomas are the most common primary intracranial tumors, accounting for approximately 81% of all malignant brain tumors (1). Although gliomas have a low incidence rate of approximately 3–8 per 100,000, their mortality rate is extremely high. Among glioma patients, adult-type diffuse gliomas are the predominant pathological type (2). Gliomas are known to be genetically heterogeneous and complex (3), making it difficult to predict the outcome of gliomas due to their rapid progression and high level of heterogeneity, even with the same pathological diagnosis and World Health Organization (WHO) grade of the tumor. Prior to the 2016 WHO classification of central nervous system (CNS) tumors, pathologists primarily relied on the under-microscope histologic features of the tumor to classify and grade the lesions. The 2016 version of the CNS tumor classification introduced the classification of gliomas based on the coexistence of histologic and molecular features of the tumor, and incorporated molecular information such as IDH, 1p19q, among others, to grade and diagnose gliomas (4). This assists neurosurgeons and oncologists in predicting outcomes and developing individualized treatment strategies for different patients. However, this classification is still limited to specific molecules, such as IDH, and predicting the patient’s prognosis remains challenging. Currently, the WHO CNS5 (2021) has emphasized the importance of molecular biomarkers in providing accurate diagnostic and therapeutic information for gliomas (5). Enrichment strategies using precise biomarkers will help improve the current glioma treatment dilemma. With the development of molecular sequencing technology and increasing research progress on the correlation between different molecules and the classification of gliomas, the identification of molecular information, such as CDKN2A/B co-deletion, EGFR amplification, TERT promoter mutations, and 1p/19q co-deletion, has allowed physicians to make a more accurate individual diagnosis and assessment of prognosis for patients (6). Despite an increasing number of molecules being detected in tumor tissues, many potential prognostic markers remain to be explored. It is urgent to identify prognosis-related molecules in gliomas and integrate these molecular markers into a quantitative, specific risk score.

Machine learning broadly refers to the process of fitting a predictive model to the data or identifying groupings of information within the data, which can replace the investigator’s process of mechanistically repeated data analysis and is not influenced by the investigator’s subjective judgment. Machine learning algorithms can be objectively and more accurately applied in predicting tumor patient outcomes. Prognostic models based on machine learning have been widely used in predicting prognosis in some solid cancers (7–9) and diseases (10–13). However, a prognostic signature for predicting the OS of patients with glioma based on the newest WHO CNS5 biomarkers has not yet been reported.

In this study, we examined the profiles of around 60 WHO CNS5-related molecules in 198 glioma patients from our hospital. Multiple machine learning models were applied to identify the most important prognostic indicators among glioma patients. Additionally, we aimed to design a risk signature for predicting the OS rate using machine learning algorithms. Finally, we deployed the best performing model as an online calculator to provide an interactive, online and graphical representation of personalized survival assessment, promoting the reproducibility of the current research and external verification and implementation of the development model.

## Methods

### Study population and data collection

This retrospective study analyzed collected data from individuals who were hospitalized in our hospital from January 2011 to April 2022. A total of 204 hospitalized glioma patients were collected and randomly divided into a test set and a validation set in a ratio of 7/3. The Transparent Reporting of a Multivariable Prediction Model for Individual Prognosis or Diagnosis (TRIPOD) statement was used to report this study (14). The study included patients who underwent surgery for a histopathologically confirmed diagnosis of primary glioma. The surgery was performed by the same neurosurgeon with over 10 years of professional experience who formulated the operation plan, performed preoperative positioning, and assisted with intraoperative maximum safe range resection. All patients received standard glioma treatment, including concurrent radiotherapy and chemotherapy, adjuvant chemotherapy, and tumor treating fields technology (TTFields), based on the postoperative pathological diagnosis. Patients who died during the direct postoperative period (≤15 days after surgery) were excluded from the analysis. The study obtained ethics approval from the ethics committee at Peking Union Medical College Hospital.

### Input features and outcome

In accordance with the WHO classification of Central Nervous System tumors in its fifth edition (WHO CNS5) published in 2021 (5), we have collected molecular information on up to 60 types of markers, including mutation and copy number variation, as well as important clinical data such as sex, age, WHO grade, and WHO CNS5 pathological diagnosis, to emphasize the significance of molecular biomarkers in providing prognosis information for glioma. Prognosis information, including survival time and survival status, was also collected for patient samples. The raw data used for the analysis is available in Supplementary Table S1. The outcome variables of the prognostic model were defined as survival time and survival status, with continuous variables including the number of overall survival years from diagnosis to death, and dichotomous variables indicating survival status, with survival denoted as 0 and death denoted as 1. The dependent variables included age at diagnosis in years, sex, WHO grade, WHO CNS5 pathological diagnosis, and WHO CNS5-related molecular information, with “alteration” noted as 1 and “no alteration” noted as 0. Independent, trained data collectors collected data on input characteristics and survival outcomes.

### Statistical analysis

In this study, cases with incomplete information were removed and the remaining cohort was randomly split into a training and test set in a 70/30 ratio. Pearson correlation analysis was conducted to assess the correlation between input features (15). Univariate Cox regression analysis was performed to identify prognostic factors, and multivariate Cox regression analysis was performed to identify independent prognostic factors. The statistical analyses were performed using R version 3.6.1 software with the “survival” and “survminer” packages. The purpose of these analyses was to determine the independent association between covariates and survival, and all covariates that were statistically significantly associated with survival were included in the predictive analysis. Kaplan-Meir (K-M) method (16) was used to generate survival curves for patients with different covariates, and log-rank test was performed to assess the significance of the survival analysis.

### Machine learning-based algorithm

In this study, we employed four machine learning algorithms, namely support vector machine (SVM) (17), random forest (RF) (18), extreme gradient boosting (XGB) (19), and generalized linear model (GLM) (20), to perform feature selection and classification. To begin, we divided the samples into two groups based on the median value of survival time for patients who had already experienced a death outcome. The variables related to survival time were then screened out by the four machine learning algorithms. All machine learning modeling was performed using the R “caret” package (21). Next, the Delong test (22) was used to compare the performance of the four different machine learning models. The optimal machine learning model and its predicted signature variables significantly associated with survival time were then selected.

### Construction of Who CNS5 (WHO5) related risk signature

Lasso regression was employed to prevent overfitting. Using the survival time, survival status, and WHO5-related biomarker data of glioma patients, a risk signature was formulated through the Lasso regression algorithm, with the penalty parameter λ chosen based on 10-fold cross-validation. The alteration status of genes and their regression coefficients were obtained based on the most suitable λ value. The risk score was calculated using the formula: Risk score = factorval (1)×coefficient-factor (1) + factorval (2)×coefficient-factor (2) + ⋯ + factorval(n) × coefficient-factor(n), where n represented the number of prognostic factors, factorval represented the assigned value of the factor, and “coefficient-factor” represented the factor’s coefficient in the risk signature.

### Nomogram construction and validation

To enhance the practical utility of the WHO5-related risk signature in predicting patient OS rates, we integrated common clinical features (age, sex, WHO5 grade, WHO5 pathological diagnosis) with the WHO5 risk score to create and validate a prognostic nomogram in accordance with nomogram guidelines (23). We used the RMS package in R software version 3.6.1 to develop the nomogram prediction. Moreover, we utilized the “shiny” package and server to build and deploy an online, interactive, graphical tool based on the overall best-performing model (24).

The model’s performance was evaluated using ROC analysis, discrimination, and calibration in the training and test sets. ROC analysis assesses the model’s ability to classify observations by plotting sensitivity versus 1-specificity (25). The area under the curve (AUC) values were categorized as follows: high accuracy (0.9 < AUC-ROC ≤1), moderate accuracy (0.7 < AUC-ROC ≤0.9), and low accuracy (0.5 < AUC-ROC ≤0.7) (26). Calibration plots were used to assess the relationship between predicted survival probability and observed survival (27). The c-index was used to quantify discrimination, ranging from 0.5–1.0, with 0.5 indicating completely non-discriminatory results and 1.0 indicating perfect discrimination (28). Decision curve analysis was utilized to assess potential decision thresholds and clinical usefulness (29).

## Results

**Figure 1:**
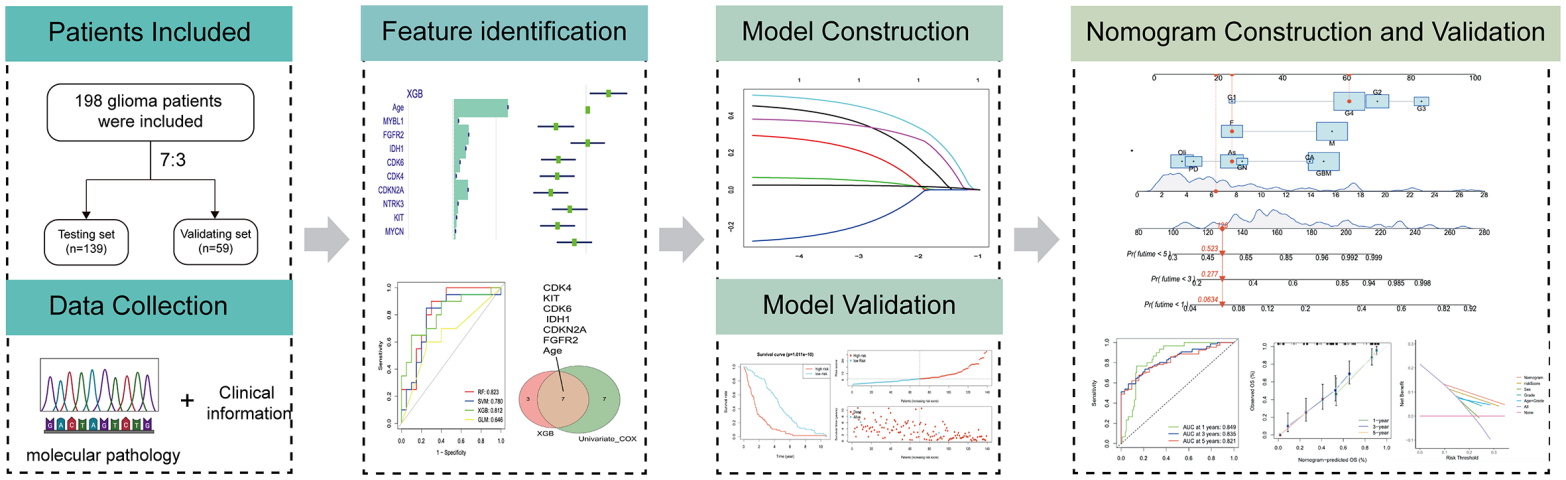
Workflow of the study.

The flow diagram in Figure 1 illustrates the study inclusion process.

### Patient demographics and clinical characteristics

This study included a total of 198 patients who had complete WHO5 molecular information and follow-up data. The median overall survival time for all patients was 29.77 months, with a 95% confidence interval of 21.19–38.34 months. The patient cohort was randomly split into a training set of 139 patients and a hold-out test set of 59 patients, and their molecular and clinical information related to WHO5 was summarized in Table 1. Among the two sets, only the sex composition ratio was significantly different (Supplementary Table S1). After removing variables with relatively small variance changes and negligible effects using the “sklearn” Library of Python 3.7, a total of 32 independent variables, including pathological diagnosis, were eligible for subsequent analysis.

**Table 1:** Clinical feature and WHO5 related information of patients with gliomas in train and test sets.

Covariates	Type	Total	Train	Test	Pvalue
Sex	F	73 (36.87%)	42 (30.22%)	31 (52.54%)	0.0048
	M	125 (63.13%)	97 (69.78%)	28 (47.46%)	
Age	<=47	174 (87.88%)	125 (89.93%)	49 (83.05%)	0.2635
	>47	24 (12.12%)	14 (10.07%)	10 (16.95%)	
WHO5_pathologic_diagnosis	Astrocytoma	34 (17.17%)	30 (21.58%)	4 (6.78%)	NA
	Circumscribed astrocytic gliomas	4 (2.02%)	2 (1.44%)	2 (3.39%)	
	Glioblastoma	88 (44.44%)	53 (38.13%)	35 (59.32%)	
	Glioneuronal and neuronal tumors	14 (7.07%)	7 (5.04%)	7 (11.86%)	
	Oligodendroglioma	42 (21.21%)	31 (22.3%)	11 (18.64%)	
	Pediatric-type diffuse gliomas	16 (8.08%)	16 (11.51%)	0 (0%)	
Grade	HGG	138 (69.7%)	92 (66.19%)	46 (77.97%)	0.1387
	LGG	60 (30.3%)	47 (33.81%)	13 (22.03%)	
ACVR1	Alt	1 (0.51%)	1 (0.72%)	0 (0%)	1
	No	197 (99.49%)	138 (99.28%)	59 (100%)	
ATRX	Alt	37 (18.69%)	25 (17.99%)	12 (20.34%)	0.8499
	No	161 (81.31%)	114 (82.01%)	47 (79.66%)	
BCOR	Alt	6 (3.03%)	4 (2.88%)	2 (3.39%)	1
	No	192 (96.97%)	135 (97.12%)	57 (96.61%)	
BRAF	Alt	114 (57.58%)	81 (58.27%)	33 (55.93%)	0.8826
	No	84 (42.42%)	58 (41.73%)	26 (44.07%)	
CDK4	Alt	98 (49.49%)	70 (50.36%)	28 (47.46%)	0.8273
	No	100 (50.51%)	69 (49.64%)	31 (52.54%)	
CDK6	Alt	119 (60.1%)	82 (58.99%)	37 (62.71%)	0.7413
	No	79 (39.9%)	57 (41.01%)	22 (37.29%)	
CDKN2A	Alt	112 (56.57%)	78 (56.12%)	34 (57.63%)	0.9684
	No	86 (43.43%)	61 (43.88%)	25 (42.37%)	
CDKN2B	Alt	128 (64.65%)	90 (64.75%)	38 (64.41%)	1
	No	70 (35.35%)	49 (35.25%)	21 (35.59%)	
CIC	Alt	31 (15.66%)	22 (15.83%)	9 (15.25%)	1
	No	167 (84.34%)	117 (84.17%)	50 (84.75%)	
EGFR	Alt	120 (60.61%)	84 (60.43%)	36 (61.02%)	1
	No	78 (39.39%)	55 (39.57%)	23 (38.98%)	
FBXW7	Alt	7 (3.54%)	4 (2.88%)	3 (5.08%)	0.7275
	No	191 (96.46%)	135 (97.12%)	56 (94.92%)	
FGFR1	Alt	76 (38.38%)	53 (38.13%)	23 (38.98%)	1
	No	122 (61.62%)	86 (61.87%)	36 (61.02%)	
FGFR2	Alt	90 (45.45%)	60 (43.17%)	30 (50.85%)	0.4027
	No	108 (54.55%)	79 (56.83%)	29 (49.15%)	
FGFR3	Alt	59 (29.8%)	40 (28.78%)	19 (32.2%)	0.7548
	No	139 (70.2%)	99 (71.22%)	40 (67.8%)	
FGFR4	Alt	57 (28.79%)	39 (28.06%)	18 (30.51%)	0.8597
	No	141 (71.21%)	100 (71.94%)	41 (69.49%)	
FUBP1	Alt	13 (6.57%)	10 (7.19%)	3 (5.08%)	0.8146
	No	185 (93.43%)	129 (92.81%)	56 (94.92%)	
H3F3A	Alt	1 (0.51%)	1 (0.72%)	0 (0%)	1
	No	197 (99.49%)	138 (99.28%)	59 (100%)	
HIST1H3B	Alt	1 (0.51%)	1 (0.72%)	0 (0%)	1
	No	197 (99.49%)	138 (99.28%)	59 (100%)	
HIST1H3C	No	198 (100%)	139 (100%)	59 (100%)	NA
IDH1	Alt	78 (39.39%)	59 (42.45%)	19 (32.2%)	0.234
	No	120 (60.61%)	80 (57.55%)	40 (67.8%)	
IDH2	Alt	2 (1.01%)	1 (0.72%)	1 (1.69%)	1
	No	196 (98.99%)	138 (99.28%)	58 (98.31%)	
KIT	Alt	67 (33.84%)	47 (33.81%)	20 (33.9%)	1
	No	131 (66.16%)	92 (66.19%)	39 (66.1%)	
KMT5B	Alt	26 (13.13%)	19 (13.67%)	7 (11.86%)	0.9094
	No	172 (86.87%)	120 (86.33%)	52 (88.14%)	
KRAS	Alt	86 (43.43%)	62 (44.6%)	24 (40.68%)	0.724
	No	112 (56.57%)	77 (55.4%)	35 (59.32%)	
MAP2K1	No	198 (100%)	139 (100%)	59 (100%)	NA
MET	Alt	78 (39.39%)	54 (38.85%)	24 (40.68%)	0.9347
	No	120 (60.61%)	85 (61.15%)	35 (59.32%)	
MYB	Alt	82 (41.41%)	58 (41.73%)	24 (40.68%)	1
	No	116 (58.59%)	81 (58.27%)	35 (59.32%)	
MYBL1	Alt	54 (27.27%)	38 (27.34%)	16 (27.12%)	1
	No	144 (72.73%)	101 (72.66%)	43 (72.88%)	
MYC	Alt	63 (31.82%)	46 (33.09%)	17 (28.81%)	0.6711
	No	135 (68.18%)	93 (66.91%)	42 (71.19%)	
MYCN	Alt	39 (19.7%)	28 (20.14%)	11 (18.64%)	0.9622
	No	159 (80.3%)	111 (79.86%)	48 (81.36%)	
NF1	Alt	16 (8.08%)	11 (7.91%)	5 (8.47%)	1
	No	182 (91.92%)	128 (92.09%)	54 (91.53%)	
NOTCH1	Alt	69 (34.85%)	51 (36.69%)	18 (30.51%)	0.5016
	No	129 (65.15%)	88 (63.31%)	41 (69.49%)	
NRAS	Alt	3 (1.52%)	2 (1.44%)	1 (1.69%)	1
	No	195 (98.48%)	137 (98.56%)	58 (98.31%)	
NTRK2	Alt	85 (42.93%)	60 (43.17%)	25 (42.37%)	1
	No	113 (57.07%)	79 (56.83%)	34 (57.63%)	
NTRK3	Alt	61 (30.81%)	43 (30.94%)	18 (30.51%)	1
	No	137 (69.19%)	96 (69.06%)	41 (69.49%)	
PDGFRA	Alt	88 (44.44%)	63 (45.32%)	25 (42.37%)	0.8213
	No	110 (55.56%)	76 (54.68%)	34 (57.63%)	
PEG3	Alt	102 (51.52%)	72 (51.8%)	30 (50.85%)	1
	No	96 (48.48%)	67 (48.2%)	29 (49.15%)	
PIK3CA	Alt	125 (63.13%)	89 (64.03%)	36 (61.02%)	0.8098
	No	73 (36.87%)	50 (35.97%)	23 (38.98%)	
PIK3CB	Alt	6 (3.03%)	4 (2.88%)	2 (3.39%)	1
	No	192 (96.97%)	135 (97.12%)	57 (96.61%)	
PIK3R1	Alt	12 (6.06%)	9 (6.47%)	3 (5.08%)	0.9607
	No	186 (93.94%)	130 (93.53%)	56 (94.92%)	
PPM1D	Alt	188 (94.95%)	132 (94.96%)	56 (94.92%)	1
	No	10 (5.05%)	7 (5.04%)	3 (5.08%)	
PTEN	Alt	127 (64.14%)	86 (61.87%)	41 (69.49%)	0.3894
	No	71 (35.86%)	53 (38.13%)	18 (30.51%)	
PTPN11	Alt	46 (23.23%)	32 (23.02%)	14 (23.73%)	1
	No	152 (76.77%)	107 (76.98%)	45 (76.27%)	
RB1	Alt	87 (43.94%)	61 (43.88%)	26 (44.07%)	1
	No	111 (56.06%)	78 (56.12%)	33 (55.93%)	
SMARCA4	Alt	8 (4.04%)	6 (4.32%)	2 (3.39%)	1
	No	190 (95.96%)	133 (95.68%)	57 (96.61%)	
SMARCB1	Alt	1 (0.51%)	1 (0.72%)	0 (0%)	1
	No	197 (99.49%)	138 (99.28%)	59 (100%)	
TERT	Alt	114 (57.58%)	79 (56.83%)	35 (59.32%)	0.8676
	No	84 (42.42%)	60 (43.17%)	24 (40.68%)	
TOP3A	Alt	75 (37.88%)	53 (38.13%)	22 (37.29%)	1
	No	123 (62.12%)	86 (61.87%)	37 (62.71%)	
TP53	Alt	60 (30.3%)	44 (31.65%)	16 (27.12%)	0.6411
	No	138 (69.7%)	95 (68.35%)	43 (72.88%)	
TSC1	Alt	1 (0.51%)	1 (0.72%)	0 (0%)	1
	No	197 (99.49%)	138 (99.28%)	59 (100%)	
TSC2	Alt	13 (6.57%)	9 (6.47%)	4 (6.78%)	1
	No	185 (93.43%)	130 (93.53%)	55 (93.22%)	
YAP1	Alt	2 (1.01%)	2 (1.44%)	0 (0%)	0.8815
	No	196 (98.99%)	137 (98.56%)	59 (100%)	
chr1p	Alt	193 (97.47%)	135 (97.12%)	58 (98.31%)	1
	No	5 (2.53%)	4 (2.88%)	1 (1.69%)	
chr7p	Alt	193 (97.47%)	135 (97.12%)	58 (98.31%)	1
	No	5 (2.53%)	4 (2.88%)	1 (1.69%)	
chr7q	Alt	183 (92.42%)	127 (91.37%)	56 (94.92%)	0.5691
	No	15 (7.58%)	12 (8.63%)	3 (5.08%)	
chr9p	Alt	195 (98.48%)	136 (97.84%)	59 (100%)	0.6163
	No	3 (1.52%)	3 (2.16%)	0 (0%)	
chr10p	Alt	192 (96.97%)	133 (95.68%)	59 (100%)	0.2431
	No	6 (3.03%)	6 (4.32%)	0 (0%)	
chr10q	Alt	195 (98.48%)	136 (97.84%)	59 (100%)	0.6163
	No	3 (1.52%)	3 (2.16%)	0 (0%)	
chr17	Alt	184 (92.93%)	128 (92.09%)	56 (94.92%)	0.6839
	No	14 (7.07%)	11 (7.91%)	3 (5.08%)	
chr19q	Alt	173 (87.37%)	121 (87.05%)	52 (88.14%)	1
	No	25 (12.63%)	18 (12.95%)	7 (11.86%)	

### Identification of prognostic factors

**Figure 2:**
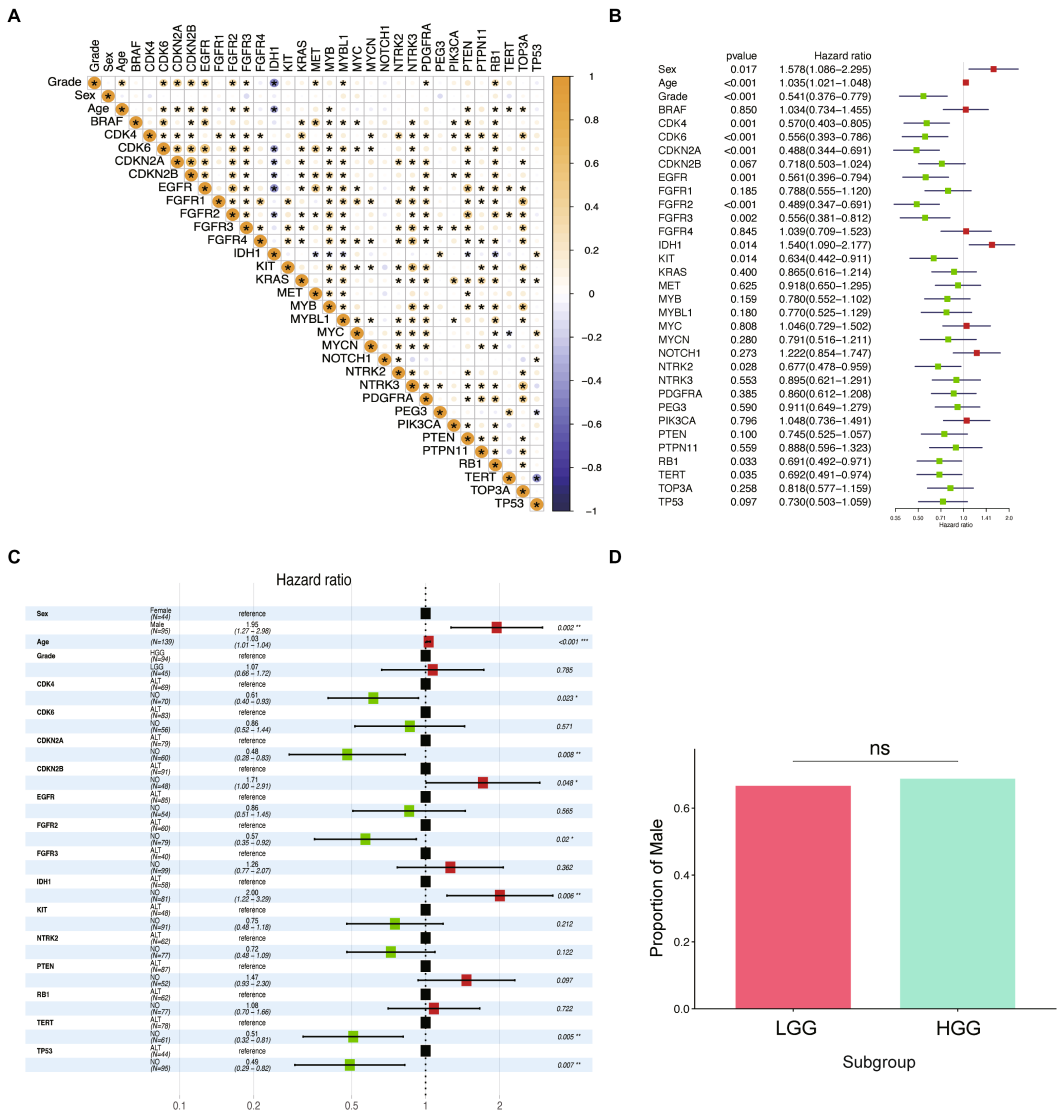
Identification of prognostic factors in glioma. **(A)** Correlation diagram of all independent variables is presented in the figure. The Pearson correlations between the independent variables used in the analysis are displayed using colors, where yellow indicates a positive correlation and blue indicates a negative correlation. The deeper the color, the stronger the correlation. An asterisk is used to denote statistical significance with a value of p of less than 0.05. **(B)** Forest plot of hazard ratios from univariate Cox regression analysis of the risk factors in glioma. Red forest plots represent risky factors, and green forest plots represent protective factors. **(C)** Forest plot of hazard ratios from multivariable Cox regression analysis of the risk factors in glioma. **(D)** Boxplot of the proportion of “Male” among LGG and HGG groups.

We conducted correlation analysis on 32 variables in the cohort of 198 participants and presented the results of Pearson’s correlation analysis in Figure 2A. Age and alterations in CDK6, CDKN2A, CDKN2B, EGFR, FGFR2, FGFR3, MET, MYBL1, PDGFRA, and RB1 were positively correlated with WHO grade (*p* < 0.05), while the alterations of IDH1 had a significant negative correlation with the patient’s grade. To investigate the prognosis-related factors in glioma, we performed univariate and multivariate Cox prognostic analyses on the molecular and clinical information. The forest plot of univariate Cox analysis indicated that 14 factors were significantly correlated with prognosis (Figure 2B). Multivariate Cox prognostic analysis revealed that age, sex, TERT, IDH1, TP53, CDKN2A, FGFR2, CDK4, and CDKN2B were independent prognostic factors in glioma patients (Figure 2C). Additionally, we performed univariate and multivariate Cox analyses on these factors in low-grade glioma (LGG) and high-grade glioma (HGG), respectively (Supplementary Table S2). Interestingly, although sex was independently associated with the overall prognosis of glioma (*p* < 0.05), we found that it was not an independent prognostic factor in the LGG and HGG subgroups. To validate the independent prognostic relevance of sex in the entire glioma sample (which may differ from the results of other studies), we investigated the differences in the proportion of males between high- and low-grade samples. However, we found no significant differences (Figure 2D). Therefore, this result excluded the prognostic relevance caused by the potential correlation between sex and WHO grade.

### Machine learning analysis for feature selecting

**Figure 3:**
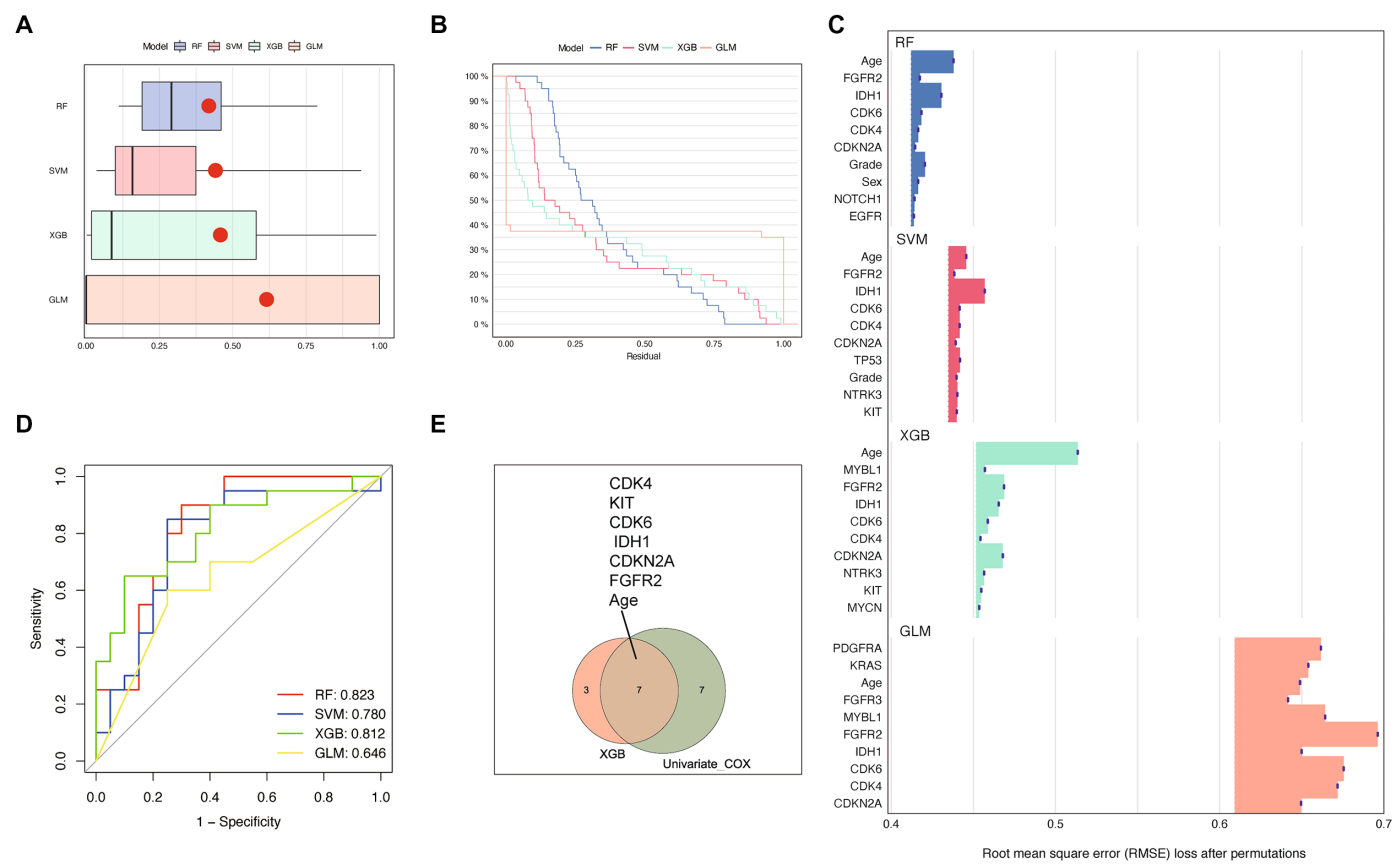
Performance comparison of prediction models based on different machine learning methods. **(A,B)** showed different residual comparisons of the four algorithms. **(A)** Each boxplot describes the residuals within an algorithm. The red dot stands for the root mean square of residuals. **(B)** Reverse cumulative distribution curves for each algorithm. **(C)** Feature importance bar charts for several machine learning algorithms. The top 10 features of each group are shown. The abscissa represents RMSE loss after permutations. RMSE, Root mean square error. **(D)** Receiver operating characteristic (ROC) curves of the four machine learning methods. **(E)** Venn diagram showing the overlapping genes of XGB and univariate cox regression analysis. The top 10 features of each group are included.

To conduct a more rigorous analysis, we utilized machine learning techniques to identify key variables associated with OS time. In order to improve the model’s performance, we employed various algorithms such as RF, SVM, XGB, and GLM as detailed in the methods section. We then evaluated the interpretability of each model on our dataset by analyzing the residual values, which are presented in Figure 3A, and the reverse cumulative distribution of residual values in Figure 3B. XGB and GLM models exhibited the smallest residual values. In Figure 3C, we demonstrated the top 10 features and their respective importance for each machine learning model. Age, FGFR2, IDH1, CDK4, CDK6, and CDKN2A were consistently identified as the most important variables. Among the models, RF and XGB had the highest ROC AUC scores in the test set, with XGB outperforming the other models (AUC 0.812 vs. AUC 0.823) as shown in Figure 3D. Finally, we combined the results of the XGB model and univariate Cox regression analysis to identify age, FGFR2, IDH1, CDK4, CDK6, KIT, and CDKN2A as crucial predictors of prognosis and OS time for glioma patients (Figure 3E). These variables were selected for further modeling of the risk signature.

### Kaplan Meier survival analysis

**Figure 4:**
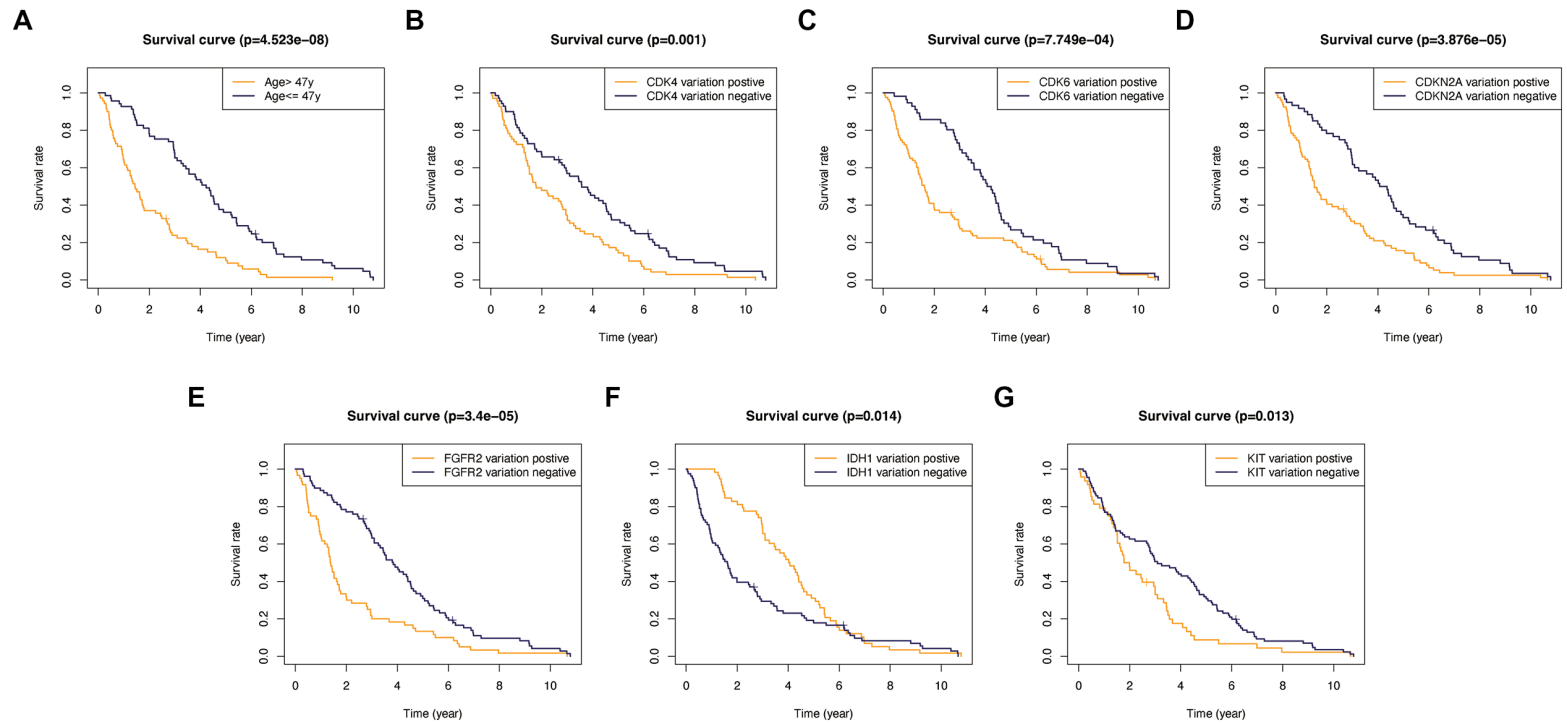
Kaplan-Meier survival plots in all glioma patients. **(A)** Age >47 vs. <=47, *p*<0.001. **(B)** CDK4 altered vs. unaltered, *p*=0.001. **(C)** CDK6 altered vs. unaltered, *p*<0.001. **(D)** CDKN2A altered vs. unaltered, *p*<0.001. **(E)** FGFR2 altered vs. unaltered, *p*<0.001. **(F)** IDH1 altered vs. unaltered, *p*=0.014. **(G)** KIT altered vs. unaltered, *p*=0.013.

**Figure 5:**
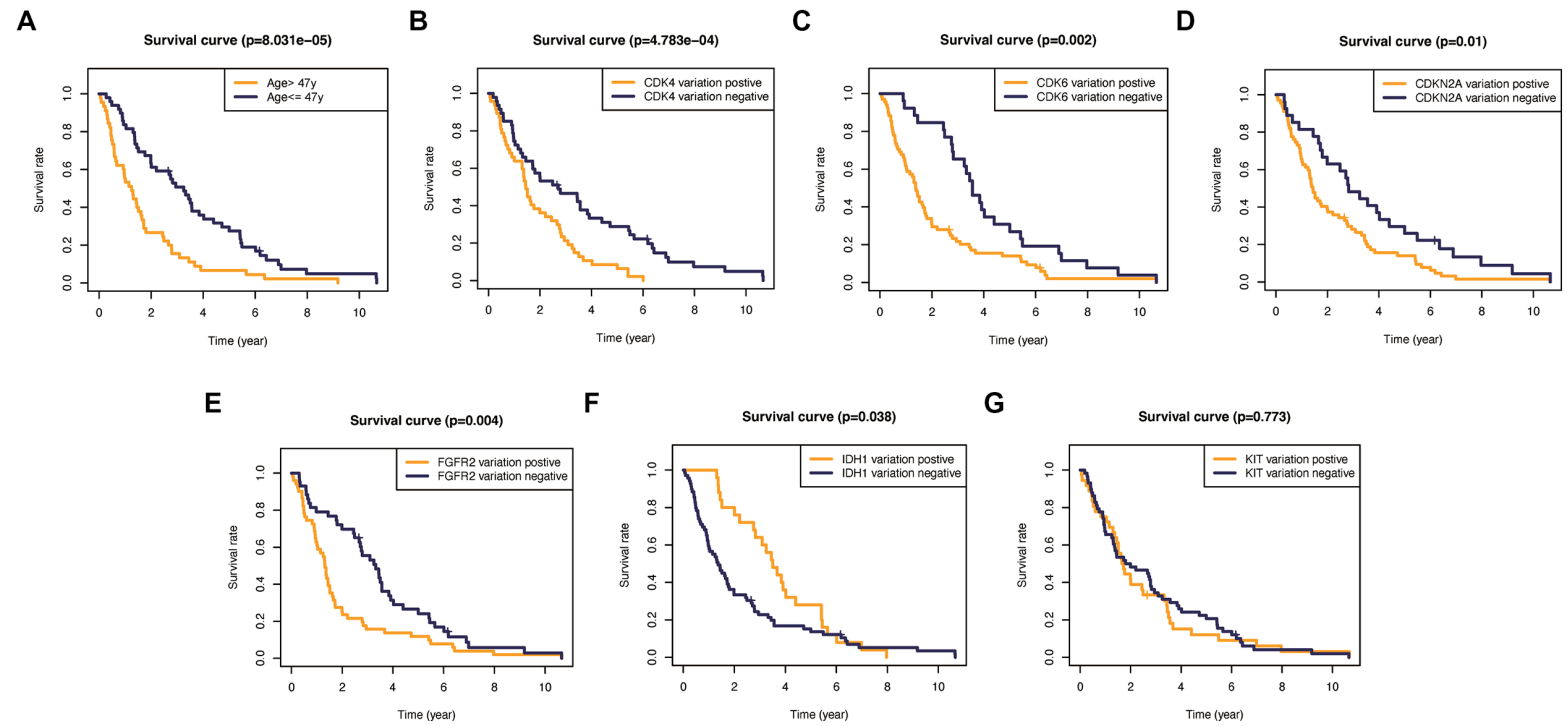
Kaplan–Meier plots of overall survival probability in patients with high-grade gliomas. **(A)** Age >47 vs. <=47, *p*<0.001. **(B)** CDK4 altered vs. unaltered, *p*<0.01. **(C)** CDK6 altered vs. unaltered, *p*=0.002. **(D)** CDKN2A altered vs. unaltered, *p*=0.01. **(E)** FGFR2 altered vs. unaltered, *p*=0.004. **(F)** IDH1 altered vs. unaltered, *p*=0.038. **(G)** KIT altered vs. unaltered, *p*=0.773.

**Figure 6:**
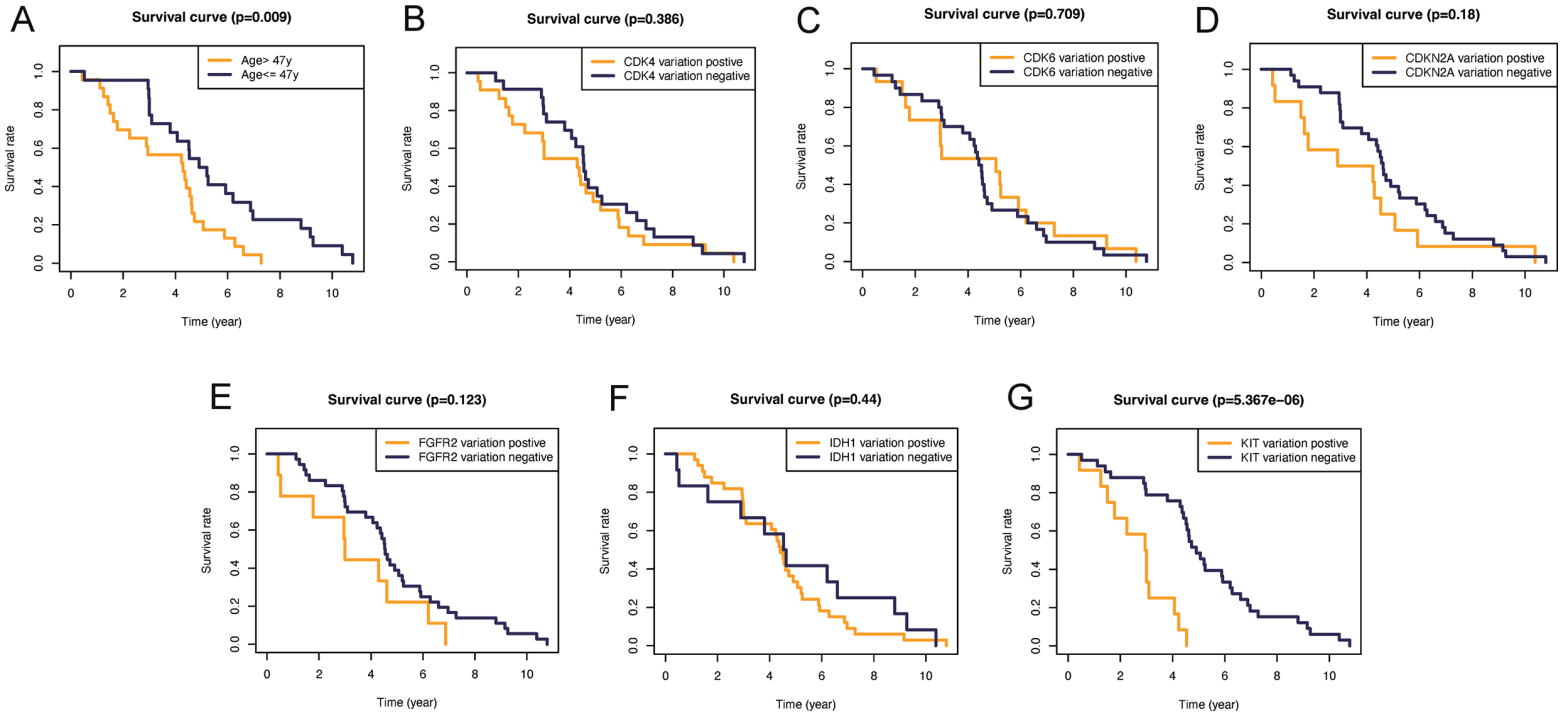
Kaplan–Meier plots of overall survival probability in patients with low-grade gliomas. Kaplan-Meier survival plots in glioma patients. **(A)** Age >47 vs. <=47, *p*=0.009. **(B)** CDK4 altered vs. unaltered, *p*=0.386. **(C)** CDK6 altered vs. unaltered, *p*=0.709. **(D)** CDKN2A altered vs. unaltered, *p*=0.18. **(E)** FGFR2 altered vs. unaltered, *p*=0.123. **(F)** IDH1 altered vs. unaltered, *p*=0.44. **(G)** KIT altered vs. unaltered, *p*<0.0001.

In addition, to validate the predictive capability of these seven factors, we generated survival curves using K-M survival analysis in the entire glioma cohort, as well as LGG and HGG subgroups. The results showed that patients in different groups defined by age, FGFR2, IDH1, CDK4, CDK6, KIT, and CDKN2A had significantly different OS in all glioma grades (Figure 4). However, in LGG samples, the alteration of KIT may not substantially distinguish the patient’s OS (Figure 5, *p* = 0.773). On the other hand, in HGG, age and KIT were found to be significantly related to the patient’s OS (Figure 6, *p* < 0.05).

### Construction of WHO5-related risk signature

**Figure 7:**
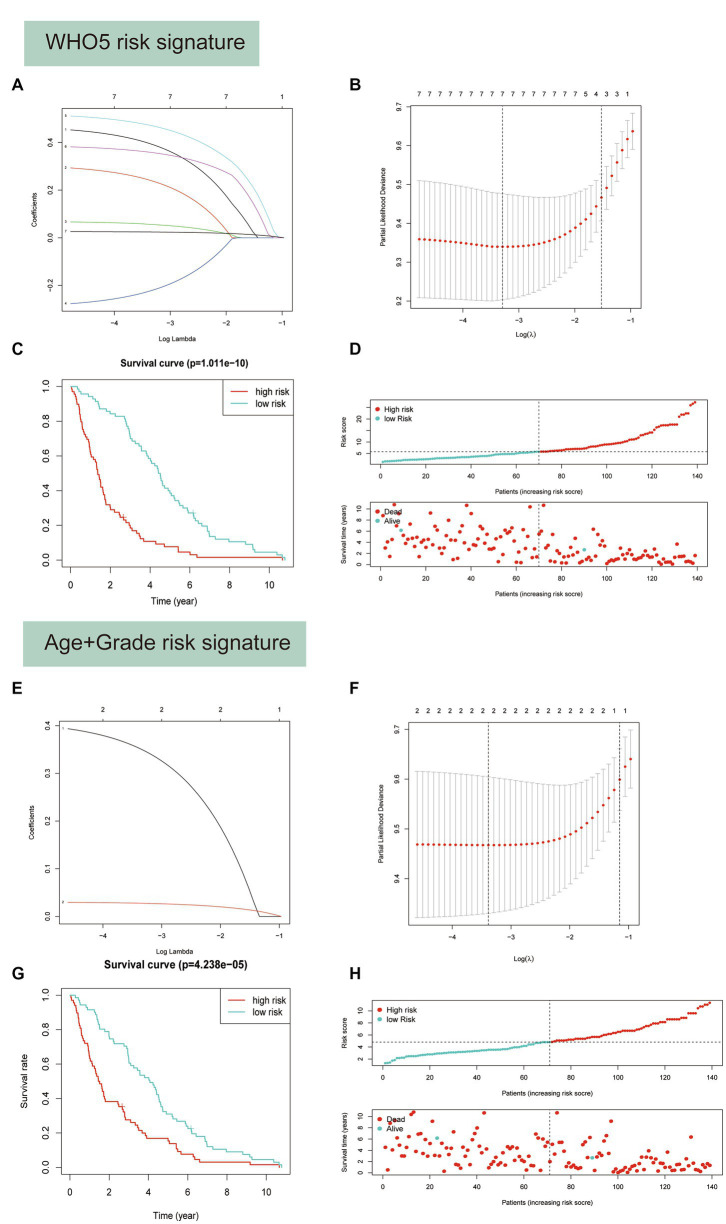
Predictor selection using the least absolute shrinkage and selection operator (LASSO) logistic regression model. **(A–D)** Construction of WHO5 risk signature scores using the LASSO regression model. **(A)** LASSO coefficient profiles of the 7 candidates. **(B)** Selection of the optimal parameter (lambda) in the LASSO model using the tenfold cross-validation. **(C)** Kaplan–Meier survival analysis for the overall survival curves of gliomas with a low or high risk of death, according the model based classifier risk score level. **(D)** The signature risk score distribution and the scatter plot of the sample survival overview in the training set. The blue and red dots, respectively, represent survival and death. **(E–H)** Construction of age and grade risk signature scores using the LASSO regression model.

Based on their prognostic relevance in glioma samples, the seven variables age, FGFR2, IDH1, CDK4, CDK6, KIT, and CDKN2A were selected for the construction of the WHO5 risk signature. The LASSO Cox regression method was used to select the most important variables and construct the signature. When log(λ) = −3.3, the seven variables were selected and used to generate risk scores for each patient, based on their alteration status (mutation or CNV is recorded as 1, and no alteration is recorded as 0) and the risk coefficient of each factor. The risk score and coefficient for each factor are shown in Supplementary Table S3, and the calculation formula is described in the methods section (Figures 7A,B).

Survival analysis showed a strong correlation between risk score and OS of patients with glioma in the training set (Figure 7C). The distributions of risk score and survival status were also plotted (Figure 7D). Additionally, a control risk model based on age and grade was constructed and had a good prognosis prediction effect in glioma patients (Figures 7E–H).

**Figure 8:**
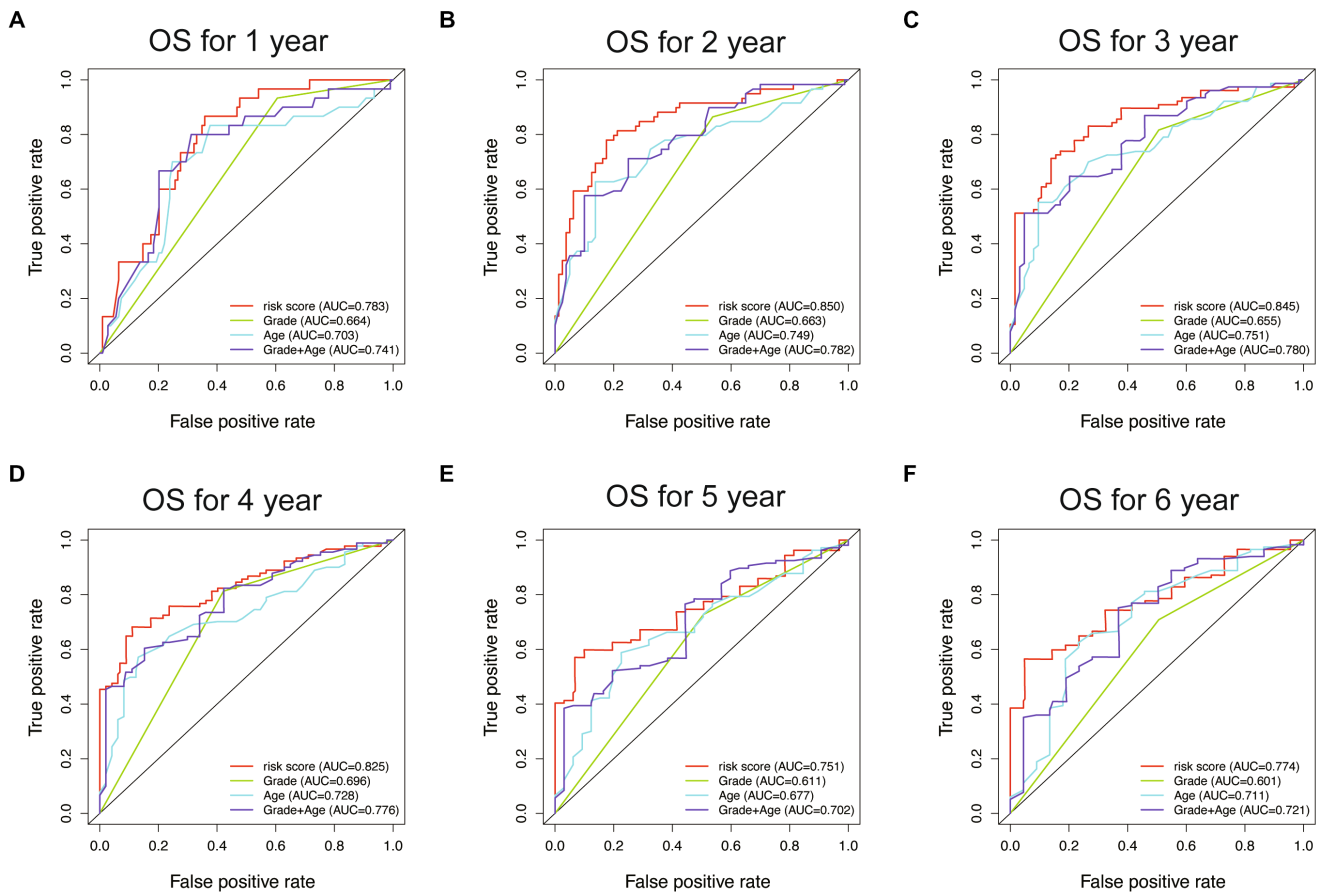
The ROC curves for the WHO5 risk score, grade, age, and control risk model in the training cohort. **(A)** ROC curve of 1-year overall survival. **(B)** ROC curve of 2-year overall survival. **(C)** ROC curve of 3-year overall survival. **(D)** ROC curve of 4-year overall survival. **(E)** ROC curve of 5-year overall survival. **(F)** ROC curve of 6-year overall survival.

To evaluate the performance of different models, receiver operating characteristic (ROC) curves were drawn in both the training and testing sets. The AUC values of the ROC curves reflect the sensitivity and specificity for predicting OS of the risk score and other clinical factors. As shown in Figure 8, the WHO5 risk signature consistently outperformed the control risk model, age, and grade in terms of AUC for predicting 1–6 years OS. The better prognostic prediction ability of the WHO5 risk signature was further validated in the testing set (Supplementary Figure S1).

### Construction and validation of the OS nomogram

**Figure 9:**
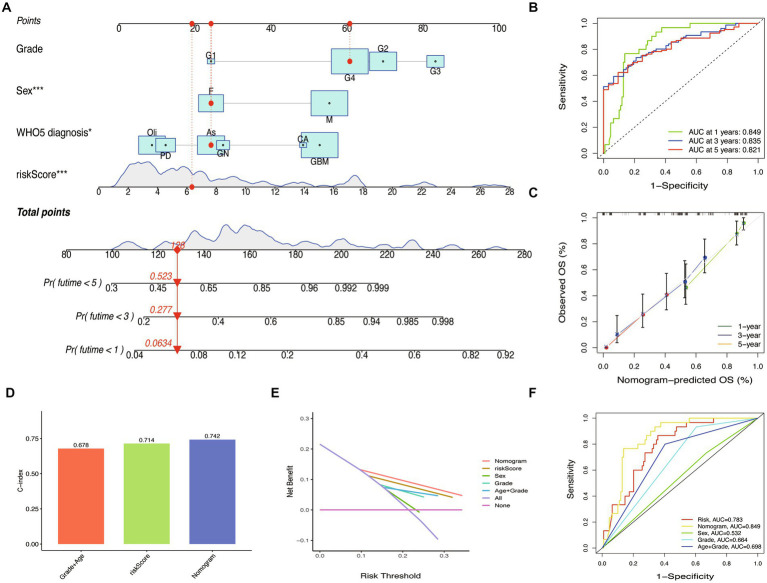
Nomogram construction and validation. **(A)** Prediction nomogram integrated the predictors selected, including grade, sex, and WHO5 diagnosis. **(B)** ROC curve of the nomogram. **(C)** Calibration curves of the nomogram. **(D)** The c-index of the control model, WHO5 risk model, and nomogram. **(E)** Decision curve analysis for different models. **(F)** ROC curves of different models.

To improve the prognostic prediction of glioma patients, we constructed a nomogram combining the riskScore with other clinical characteristics (Figure 9A). The efficiency of the nomogram was evaluated using ROC curves, and the AUC values were 0.849, 0.835, and 0.821 for predicting 1, 3, and 5-year OS (Figure 9B). The calibration plot confirmed the reliability of the nomogram (Figure 9C). Interestingly, while the riskScore had a significant classification of OS, the c-index was lower than the nomogram (WHO5 riskScore, 0.714; age combined grade, 0.678; nomogram, 0.742) (Figure 9D). In the test set, the nomogram also outperformed other scoring systems in ROC curves, calibration plots, and c-index (Supplementary Figures S2A–D). Furthermore, in the decision curve analysis, the nomogram showed a superior net benefit across a broader scale of threshold probabilities for predicting 1-, 3-, and 5-year OS than other risk factors in both training and test sets (Figure 9E; Supplementary Figure S2E). The authors concluded that by integrating the prognostic WHO5-related molecular factors into the riskScore and then combining it with other clinical-associated features, the nomogram outperformed the control risk signature and common clinical factors in terms of interpretability, predictive applicability, and computational efficiency (Figure 9F). Therefore, the authors selected it as the backend for the online survival prediction tool (Glioma Survival Calculator),^1^ which collects information on WHO5 riskScore, sex, grade, and WHO5 pathological diagnosis and calculates the survival probability for a specific time (year) and draws the survival curves of glioma patients.

## Discussion

As molecular testing advances, the integration of molecular and histological features into diagnosis and prognosis indicators in the WHO CNS5 classification has improved our understanding of the molecular categorization of gliomas (5, 6). However, the growing volumes of genomic and epigenomic data generated by high-throughput technologies present significant computational limitations for traditional statistical techniques. To overcome these constraints, modern techniques such as machine learning and data mining have been employed (30, 31). Machine learning is a subfield of artificial intelligence research that develops and evaluates algorithms to enhance pattern recognition, classification, and prediction (32). It employs various statistical, probability, and optimization methods to “learn” patterns from large, complex, or noisy data sets, and then applies this learning to categorize new data, uncover fresh patterns, or foresee future trends (33). In this study, we utilized four machine learning models, including RF, SVM, XGB, and GLM, to investigate the relationship between overall survival and the parameters of clinical presentations, pathological characteristics, and molecular alterations of gliomas, and evaluated their accuracies and performances. Our goal was to establish a superior machine learning model that comprehensively integrates the parameters of the clinical and molecular characteristics of glioma, particularly incorporating novel molecules referred to in the fifth edition of the WHO Classification of Tumors of the Central Nervous System.

In our study, we enrolled 198 patients who had undergone surgical treatment and received postoperative adjuvant radiotherapy and chemotherapy, based on their pathological diagnosis. We randomly divided the patient samples into test and verification groups using a 7:3 ratio. Our prognosis analysis revealed that age, grade, CDK4, CDK6, CDKN2A, and FGFR2 were significantly associated with overall survival. Among these factors, sex was a significant independent prognostic factor. We also excluded any potential relevance between sex and other factors in our study. The observed gender differences may be attributed to the protective effects of estrogen, the detrimental effects of testosterone, and the upregulation of androgen receptors on glioma. Additionally, host variables such as a less effective immune system may also contribute to gender differences (34–37).

We utilized machine learning techniques to evaluate markers and parameters related to patient overall survival. We applied all machine learning models to filter glioma-specific factors and chose the XGB model as the best-established one, which we used to intersect with multivariate analysis from COX. In our cohort, we discovered that age and alteration of IDH1, CDK4/6, KIT, CDKN2A, and FGFR2 were the characteristic variables associated with OS in patients. Among these genes, IDH-mutant gliomas differed fundamentally from IDH-wildtype gliomas in terms of metabolism, epigenetics, biological behavior, aggressive invasion, susceptible population, and responsiveness to therapy (38–41). CDK4 and CDK6, which regulate the cell cycle, played an important role in glioma pathogenesis (42). *KIT*, a class III receptor tyrosine kinase (RTK), was frequently involved in tumorigenic processes (43). CDKN2A homozygous deletion was a robust adverse prognosis factor in diffuse malignant IDH-mutant gliomas (44). And a decrease or loss of FGFR2 in high-grade gliomas was correlated with poor prognosis (45). We investigated the correlations between these seven variables and OS in different grade gliomas and found that elderly age was linked to poor prognosis in both high-grade gliomas (HGG) and low-grade gliomas (LGG). Moreover, alteration of CDK4/6, CDKN2A, FGFR2, and IDH1 were substantially related to inferior OS in HGG, while only KIT variation was associated with poor prognosis in LGG.

Subsequently, in order to develop a more accurate prognostic signature, we used the characteristic variables selected by machine learning to establish a LASSO regression model and generate risk scores. Our results showed significant improvements in accuracy and sensitivity compared to previous models. To further enhance the clinical utility of our findings, we integrated the WHO5 riskScore with other relevant clinical data to create an online nomogram that allows for the calculation of survival probability over a specific time frame (year). This innovative tool has practical significance in quantitatively evaluating the prognosis of glioma patients in clinical practice, as it provides more precise and individualized prognostic information. Traditional prognostic indicators and clinical experience may not always provide surgeons with accurate advice or enable patients to fully understand their conditions. To our knowledge, this is the first study to combine WHO5-related markers and other clinical data using machine learning to construct an online calculator (see text footnote 1) for glioma prognosis prediction.

Nevertheless, it is important to acknowledge that this study has some limitations associated with its retrospective design and data collection. The generalizability of the findings may be limited as the hospital is a tertiary referral center with inherent selection and referral bias. There is the potential bias in the final prediction results due to the limited age structure and population distribution in our study. Furthermore, it is worth noting that developing nations may have solid socio-economic disparities in terms of standard of care and healthcare access. Another limitation is the small sample size in the test cohort. Therefore, future studies should be conducted in a prospective, multi-central manner with larger sample sizes to validate our results. We will also consider incorporating data (external datasets or real-world cases) from other databases and pathogenic factors to enhance the prediction model.

## Conclusion

We have thoroughly reviewed the online prognosis predictor generated and validated in our study, and it represents a promising tool for guiding therapy decisions and improving the accuracy of prognosis assessment.

## Data availability statement

The original contributions presented in the study are included in the article/Supplementary materials, further inquiries can be directed to the corresponding authors.

## Ethics statement

The studies involving human participants were reviewed and approved by the ethics committee at Peking Union Medical College Hospital (2022-PUMCH-B-113). The patients/participants provided their written informed consent to participate in this study. Written informed consent was obtained from the individual(s) for the publication of any potentially identifiable images or data included in this article.

## Author contributions

LG, YW, and WM contributed to the conception and design of this study. LG, ZZ, XZ, HX, XG, and WC contributed to the analysis and interpretation of data. YNW, YKW, TL, HW, YL, YS, and other authors contribute to the WHO5 CNS data and clinical information collection. All authors read and approved the final manuscript.

## Funding

This work was funded by the Beijing Municipal Natural Science Foundation (7202150) and the National High Level Hospital Clinical Research Funding (2022-PUMCH-A-019) for YW, the National High Level Hospital Clinical Research Funding (2022-PUMCH-B-113), the Tsinghua University-Peking Union Medical College Hospital Initiative Scientific Research Program (2019ZLH101), and the Beijing Municipal Natural Science Foundation (19JCZDJC64200[Z]) for WM.

## Conflict of interest

The authors declare that the research was conducted in the absence of any commercial or financial relationships that could be construed as a potential conflict of interest.

## Publisher’s note

All claims expressed in this article are solely those of the authors and do not necessarily represent those of their affiliated organizations, or those of the publisher, the editors and the reviewers. Any product that may be evaluated in this article, or claim that may be made by its manufacturer, is not guaranteed or endorsed by the publisher.

## Footnotes

^1^ https://who5pumch.shinyapps.io/DynNomapp/

## Abbreviations

WHO: World Health Organization
CNS: Central nervous system
TRIPOD: The transparent reporting of a multivariable prediction model for Individual Prognosis or Diagnosis
WHO CNS5: The fifth edition of the WHO classification of Central Nervous System
SVM: Support vector machine
RF: Random forest
XGB: Extreme gradient boosting
GLM: Generalized linear model
OS: Overall survival
Lasso: The least absolute shrinkage and selection operator
