# Supplementary figures and images for: An online survival predictor in glioma patients using machine learning based on WHO CNS5 data
Source: Front Neurol. 2023 May 19;14:1179761. doi: 10.3389/fneur.2023.1179761 (PMC10237015; doi:10.3389/fneur.2023.1179761)

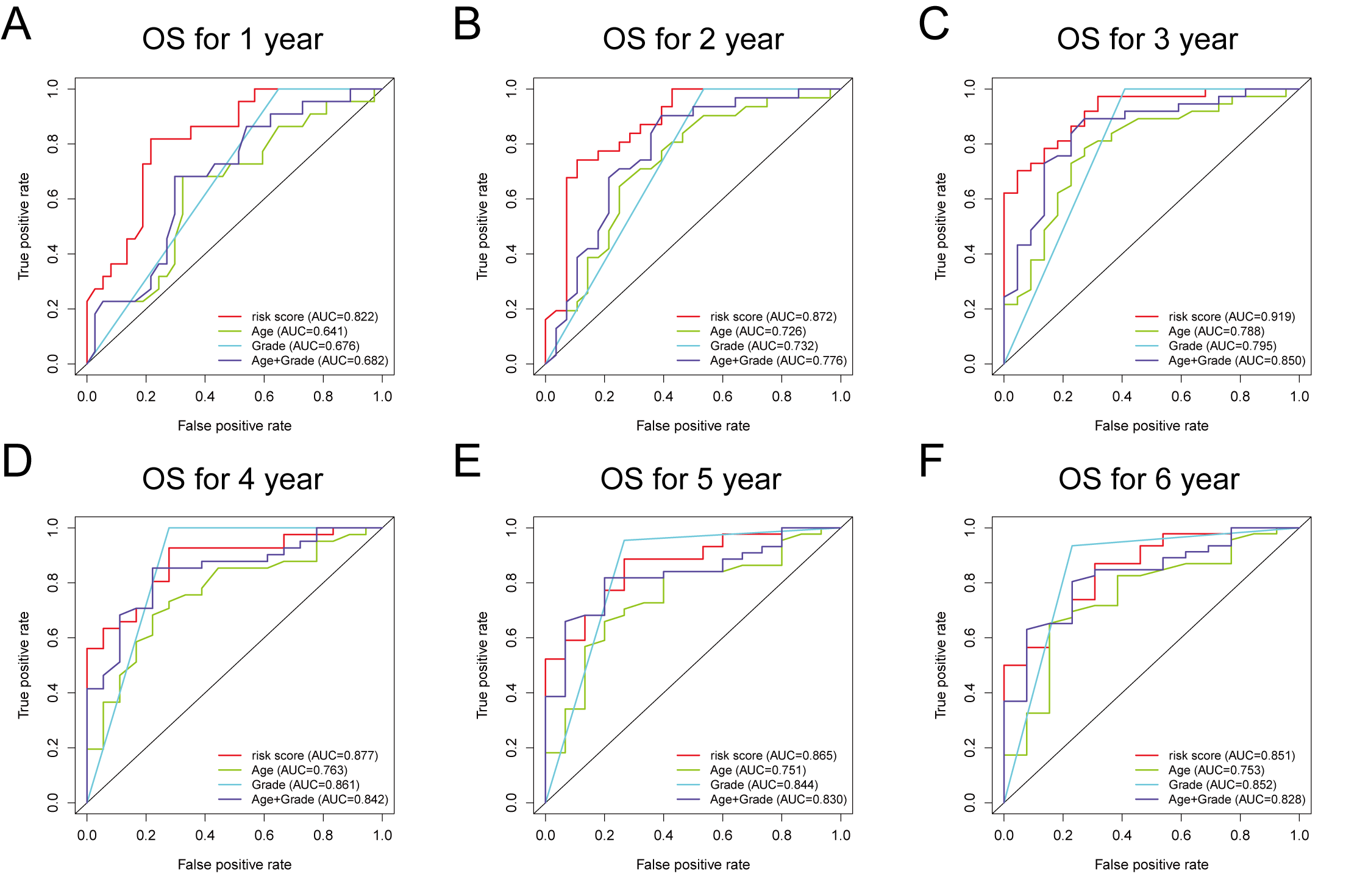

Supplement: Supplementary file 1 [file Image_1.TIF]

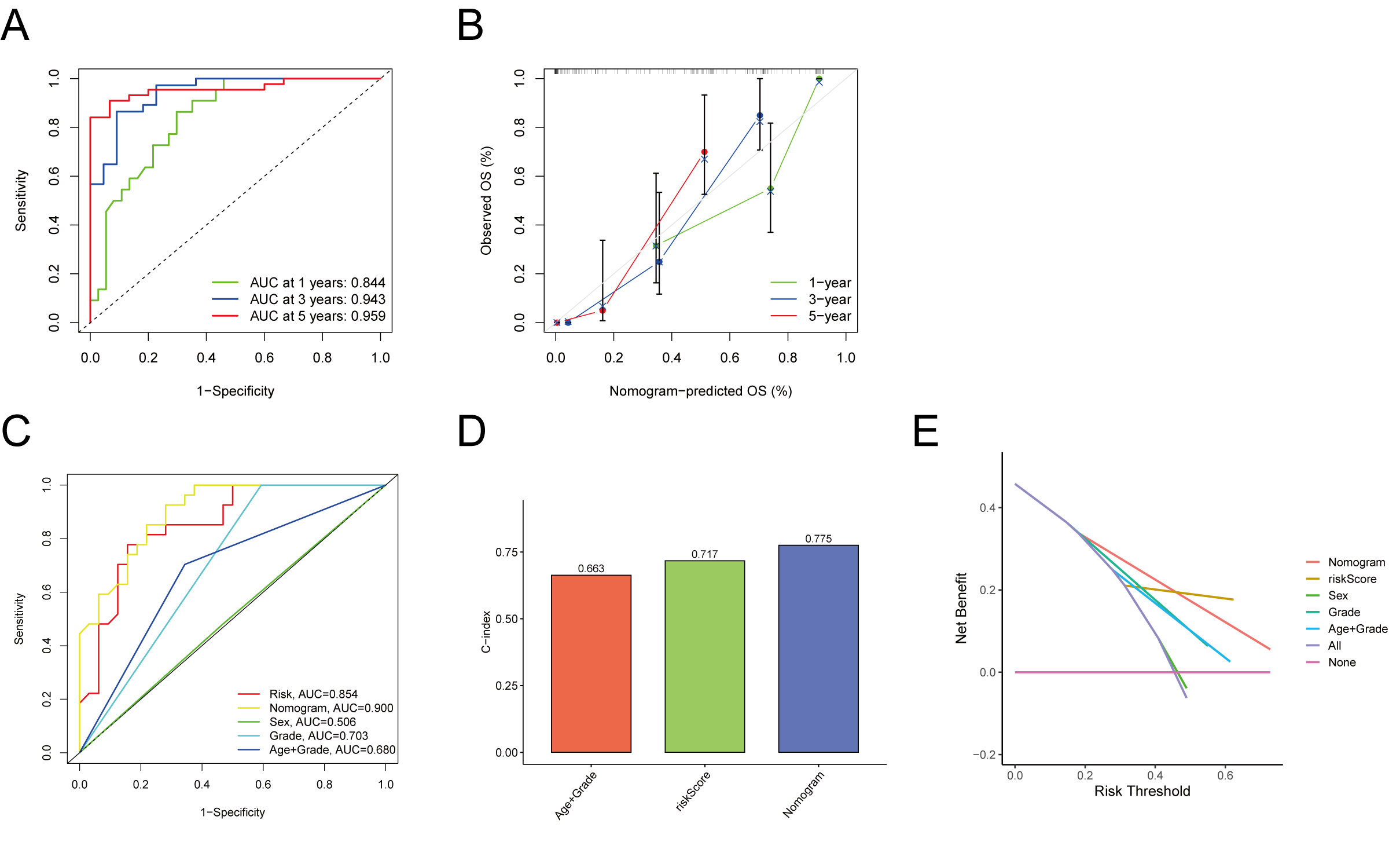

Supplement: Supplementary file 2 [file Image_2.TIF]
